# Anesthesia for Emergency Rigid Bronchoscopy in a Patient With Profound Hearing-Speech Impairment and Near-Total Tracheal Stenosis: A Case Report

**DOI:** 10.7759/cureus.117460

**Published:** 2026-10-05

**Authors:** Xiancun Liu, Xuejiao Chen, Ximing Li

**Affiliations:** 1 Anesthesiology, Linyi People's Hospital, Shandong Second Medical University, Linyi, CHN

**Keywords:** airway management, difficult airway management, rigid bronchoscopy and thoracoscopy, tracheal adenoid cystic carcinoma, tracheal stenosis

## Abstract

Primary tracheal squamous cell carcinoma producing near‑total fixed tracheal stenosis constitutes a catastrophic difficult airway scenario for emergency rigid bronchoscopy. Deaf‑mute patients face added risks of agitation, tumor hemorrhage, and airway collapse due to complete loss of communication. We implemented a pragmatic two‑stage sedation‑ventilation strategy using spontaneous breath preservation, stratified topical airway anesthesia, and tailored high‑frequency jet ventilation. This case shows that multimodal anesthesia allows safe rigid bronchoscopy in this high‑risk group. Balancing spontaneous breath preservation, topical anesthesia, and stepwise sedation is critical to avert life‑threatening airway complications. This approach yields favorable perioperative outcomes for comparable central airway pathology.

## Introduction

Near-total fixed tracheal stenosis from primary tracheal squamous cell carcinoma constitutes a life-threatening difficult airway. Severe luminal narrowing impairs respiratory reserve and causes biphasic stridor, orthopnea, cyanosis, and impending respiratory failure. Minor insults such as mucosal edema, tumor bleeding, or patient agitation may abruptly occlude the small residual airway and lead to fatal asphyxia [1,2]. Emergency rigid bronchoscopy with tumor debulking serves as definitive rescue therapy; however, perioperative anesthesia carries substantial risk given the unstable respiratory state and fragile intraluminal tumor. The patient was congenitally deaf-mute and illiterate. Without verbal, written, sign-language, or family-assisted communication, reliable patient cooperation cannot be guaranteed. This raises the risk of intraoperative agitation and unintended movement, which may trigger tumor trauma, hemorrhage, or acute airway compromise, making conventional awake airway approaches unworkable. Most existing literature describes isolated tracheal stenosis, and few reports address the combined challenge of near-total tracheal obstruction plus complete communication impairment [3]. We report a congenitally deaf-mute and illiterate patient with 96% tracheal stenosis undergoing emergency rigid bronchoscopy. This case illustrates our individualized strategy with preserved spontaneous ventilation, graded topical anesthesia, and titrated high-frequency jet ventilation (HFJV) and summarizes clinical considerations for emergent difficult airway management in patients with communication barriers.

## Case presentation

A 59‑year‑old male patient (172 cm, 68 kg) with congenital deaf‑mutism and illiteracy was admitted with intermittent hemoptysis for three days and rapidly progressive dyspnea for one day. The patient had no ability for verbal, written, or sign‑language communication, and no family members were available at the hospital during this emergency presentation. He developed progressive dyspnea accompanied by inspiratory stridor, orthopnea, and resting cyanosis and could only maintain ventilation in an upright sitting position. He had a long‑standing history of untreated hypertension and chronic bronchitis. Upon admission, his vital signs were as follows: heart rate 118 beats/min, blood pressure 145/92 mmHg, respiratory rate 32 breaths/min, and pulse oxygen saturation 82% on room air. The patient was agitated, with diffuse bilateral wheezing and crackles on lung auscultation.

Admission arterial blood gas analysis performed under 100% oxygen supplementation revealed pH 7.19, partial pressure of carbon dioxide (PaCO₂) 55 mmHg, partial pressure of oxygen (PaO₂) 141 mmHg, lactate 4.7 mmol/L, and blood glucose 16.5 mmol/L, with a hemoglobin level of 162 g/L, indicating acute type II respiratory failure and systemic tissue hypoperfusion. Laboratory examinations showed significantly elevated cardiac biomarkers: troponin T 0.173 ng/mL, creatine kinase-MB (CK‑MB) 19.60 ng/mL, myoglobin 523.00 ng/mL, and N-terminal pro-B-type natriuretic peptide (NT‑proBNP) 2333.0 pg/mL. The procalcitonin level was 9.56 ng/mL. Abnormal renal function parameters included blood urea 9.10 mmol/L and serum creatinine 127.0 μmol/L, suggesting concurrent pulmonary infection, hypoxic myocardial injury, cardiac dysfunction, and renal impairment. Chest computed tomography demonstrated a mass located in the mid‑trachea causing severe airway stenosis. The 96% tracheal luminal stenosis was quantified by measuring the minimal residual tracheal cross‑sectional area and comparing it with the estimated normal tracheal cross‑sectional area at the identical anatomical level (Figure 1). After multidisciplinary airway consultation, emergency rigid bronchoscopic tumor debulking was scheduled.

**Figure 1:**
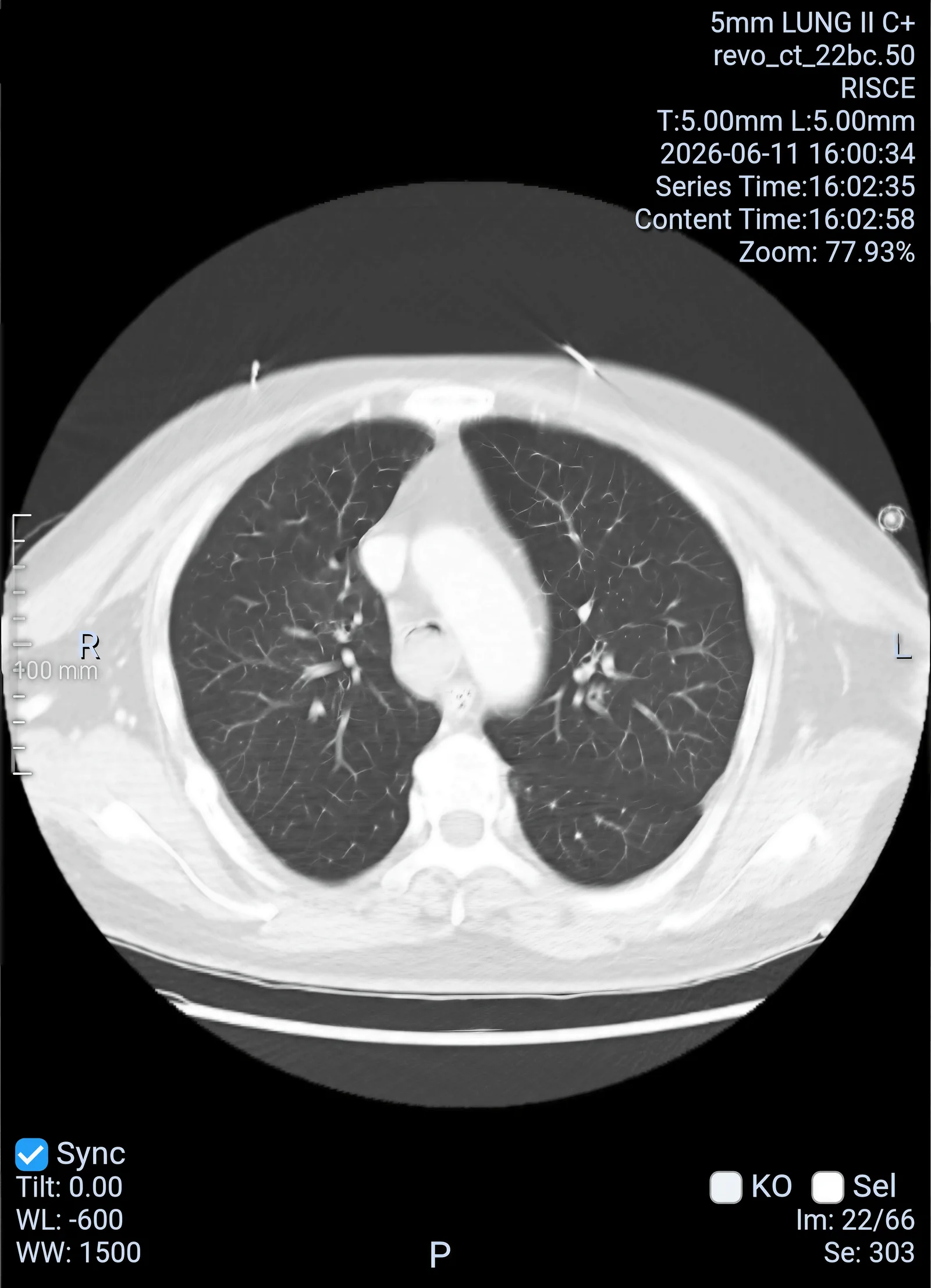
Chest computed tomography showing severe stenosis of the mid‑tracheal lumen

In the operating room, the patient was placed in a semi‑recumbent position with routine monitoring including electrocardiography, pulse oximetry, and invasive arterial blood pressure. On arrival, his vital signs were as follows: heart rate 122 beats/min, blood pressure 148/90 mmHg, respiratory rate 35 breaths/min, and oxygen saturation 92% under facial mask oxygen supplementation. Due to congenital deaf‑mutism and illiteracy, verbal, written, and sign‑language communication was impossible, and no family members were present to assist during the emergency procedure. This limited patient cooperation and increased the risk of restlessness and unplanned body movement. Considering the critical fixed tracheal stenosis, the patient's gas exchange was entirely dependent on a tiny residual airway orifice. Deep sedation or neuromuscular blockade was avoided because loss of spontaneous respiratory tone could potentially induce complete airway collapse and fatal asphyxia. Accordingly, a staged individualized anesthetic strategy was adopted to preserve spontaneous ventilation throughout the procedure without the use of muscle relaxants. Light titrated sedation was administered before airway recanalization, and sedation was gradually deepened only after airway patency was securely restored. A hierarchical multidisciplinary rescue airway protocol was established: rigid bronchoscopic tumor debulking served as the first‑line rescue intervention for complete airway obstruction or ventilation failure; emergency tracheostomy was prepared as a backup strategy, while its limitation of bypassing only supracarinal lesions was fully recognized. Veno‑venous extracorporeal membrane oxygenation (VV‑ECMO) was on standby as the final rescue option for catastrophic airway emergencies. A full difficult airway toolkit, including video laryngoscope, rigid bronchoscope, HFJV device, and tracheostomy kit, was prepared.

Oropharyngeal and base‑of‑tongue topical anesthesia was achieved using 2% lidocaine spray. Intranasal dexmedetomidine (total dose: approximately 40 μg) was administered for sedation and anxiolysis. The non‑invasive intranasal administration offers stable mucosal absorption, gradual onset, and minimal respiratory depression and does not require patient cooperation, which is uniquely suitable for patients with complete communication barriers. After topical anesthesia took effect, incremental spraying of 2% tetracaine (total dose: ≤40 mg) via flexible bronchoscopy was performed to reinforce tracheal mucosal anesthesia. Bronchoscopic exploration identified a large, friable, highly vascular tracheal mass adjacent to the carina, which precluded the passage of a 2.8 mm flexible bronchoscope. Given the tumor's bleeding tendency and severe luminal narrowing, the procedure was converted to rigid bronchoscopic tumor resection (Figure 2).

**Figure 2:**
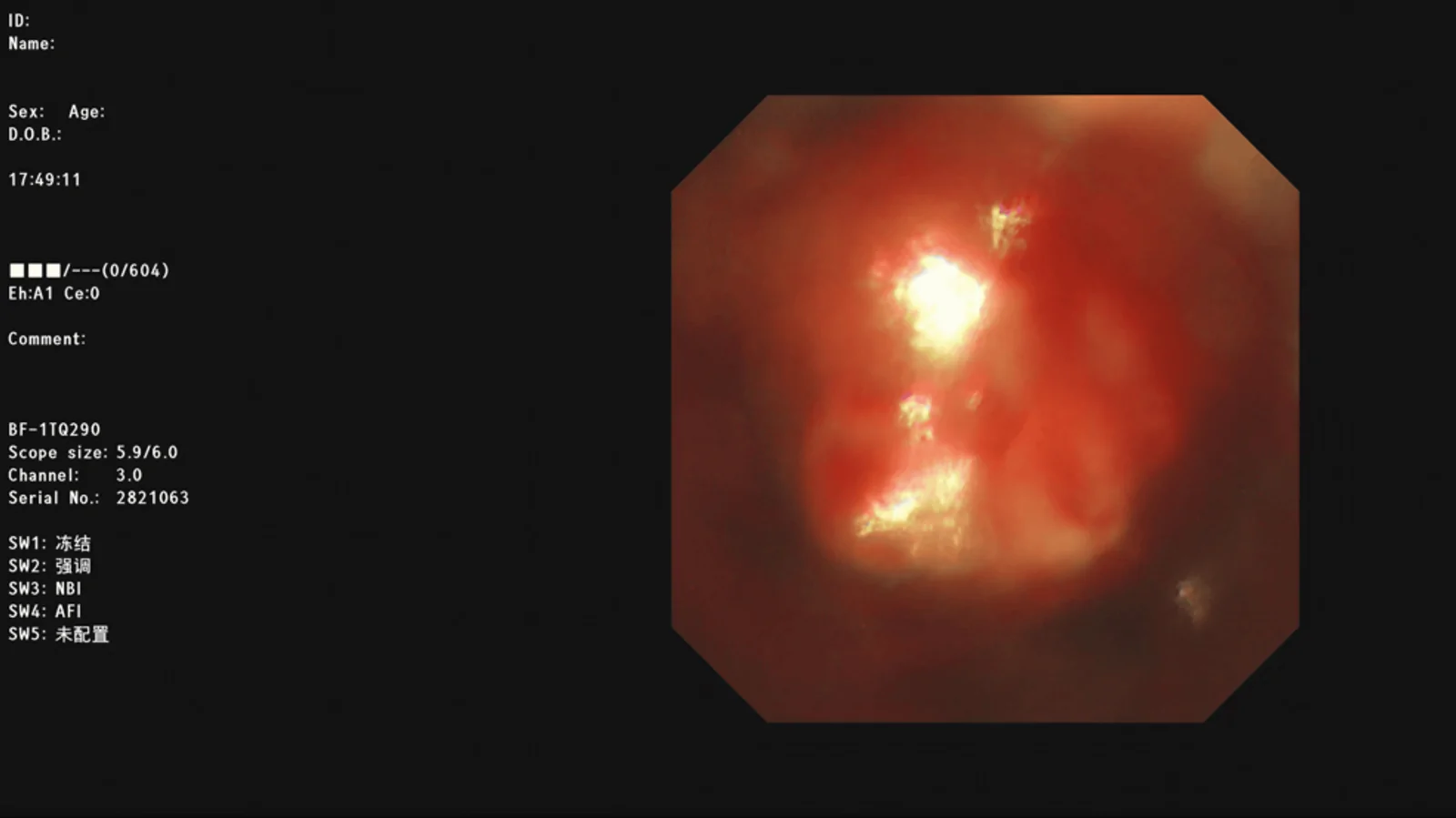
Bronchoscopic view showing the tracheal mass occupying the lumen, which was nearly occluded

Spontaneous ventilation was strictly maintained during the entire procedure. During sevoflurane inhalational induction and HFJV delivery, spontaneous breathing was continuously monitored by observation of chest wall movement, auscultation of breath sounds, and respiratory rate. No apnea or hypoventilation was detected throughout this phase. Inhalational anesthesia induction was performed with 7% sevoflurane in 100% oxygen for two minutes. After the patient's body movement stabilized, a rigid bronchoscope was inserted, and HFJV was initiated. The jet catheter was positioned at the mid‑shaft of the bronchoscope. The bronchoscopist directly confirmed the existence of a patent expiratory channel under endoscopic vision before activating HFJV. HFJV parameters were set at a driving pressure of 0.25 MPa, a ventilation frequency of 60 cycles/min, and an inspiratory‑to‑expiratory ratio of 1:1.5. Notably, real‑time monitoring of airway pressure and end‑tidal carbon dioxide was not available during HFJV due to technical limitations of this emergency setup.

Prior to argon plasma coagulation, the inspired oxygen fraction was reduced to 0.21, and oxygen supply was suspended for 60 seconds before activating the electrosurgical energy source to mitigate the risk of airway fire. Direct intraoperative observation confirmed 96% tracheal luminal stenosis, consistent with preoperative CT quantification. No direct measurement of intraluminal oxygen concentration was performed intraoperatively. Most intraluminal tumor tissue was resected using a snare and biopsy forceps, and hemostasis was achieved via electrocoagulation, topical hemocoagulase application, and iced saline irrigation (Figure 3). After adequate airway decompression and significant restoration of airway patency, a 10 mg bolus of remimazolam was administered, followed by continuous intravenous infusion of remimazolam at 0.15 mg·kg⁻¹·h⁻¹ and remifentanil at 0.10 μg·kg⁻¹·min⁻¹. Staged sedation deepening was safely implemented under secured airway conditions to reduce surgical stress and suppress involuntary patient movement. The finally resected tumor specimen measured approximately 5 cm in longitudinal length (Figure 4).

**Figure 3:**
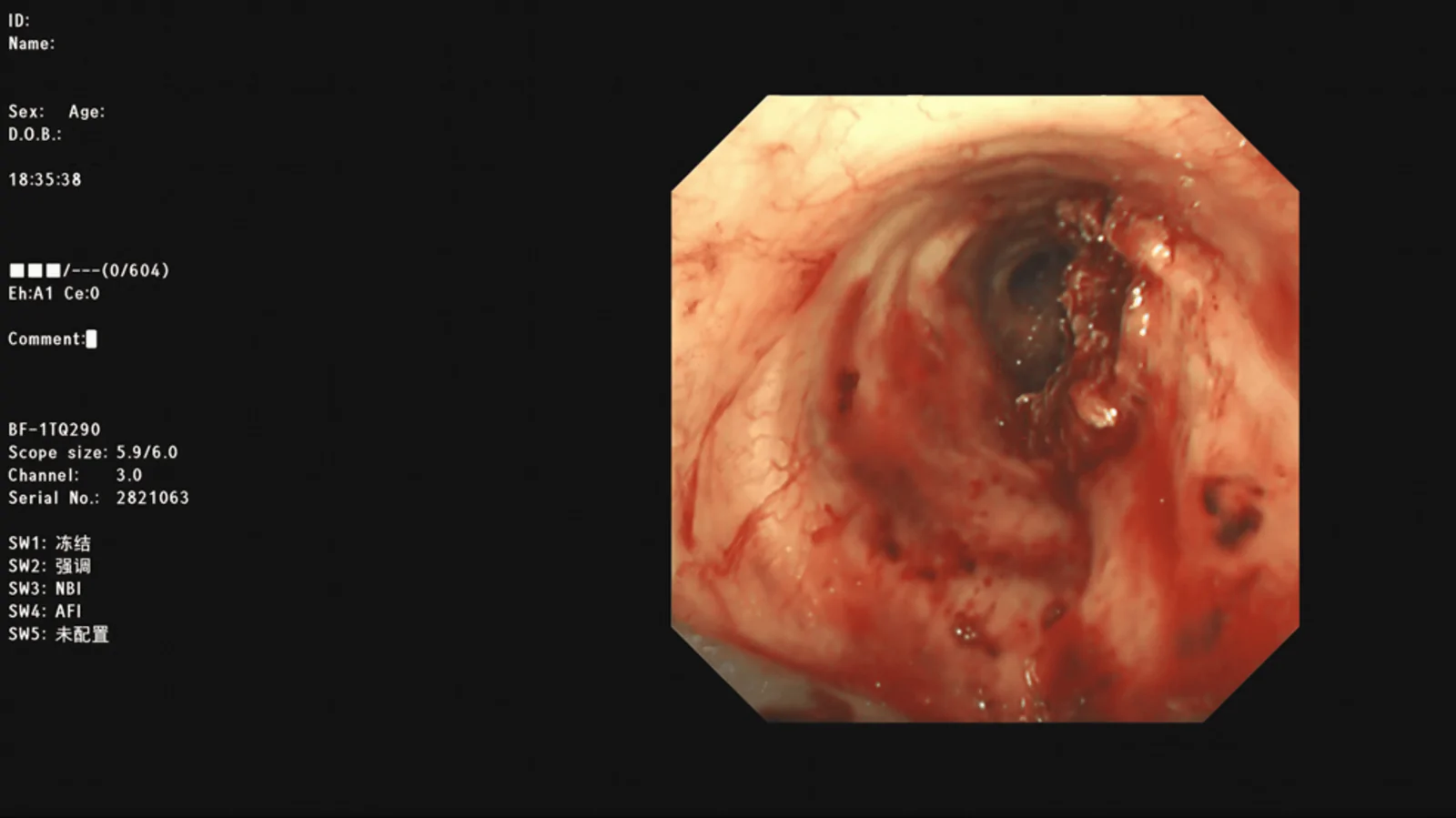
Bronchoscopic view after partial tumor resection

**Figure 4:**
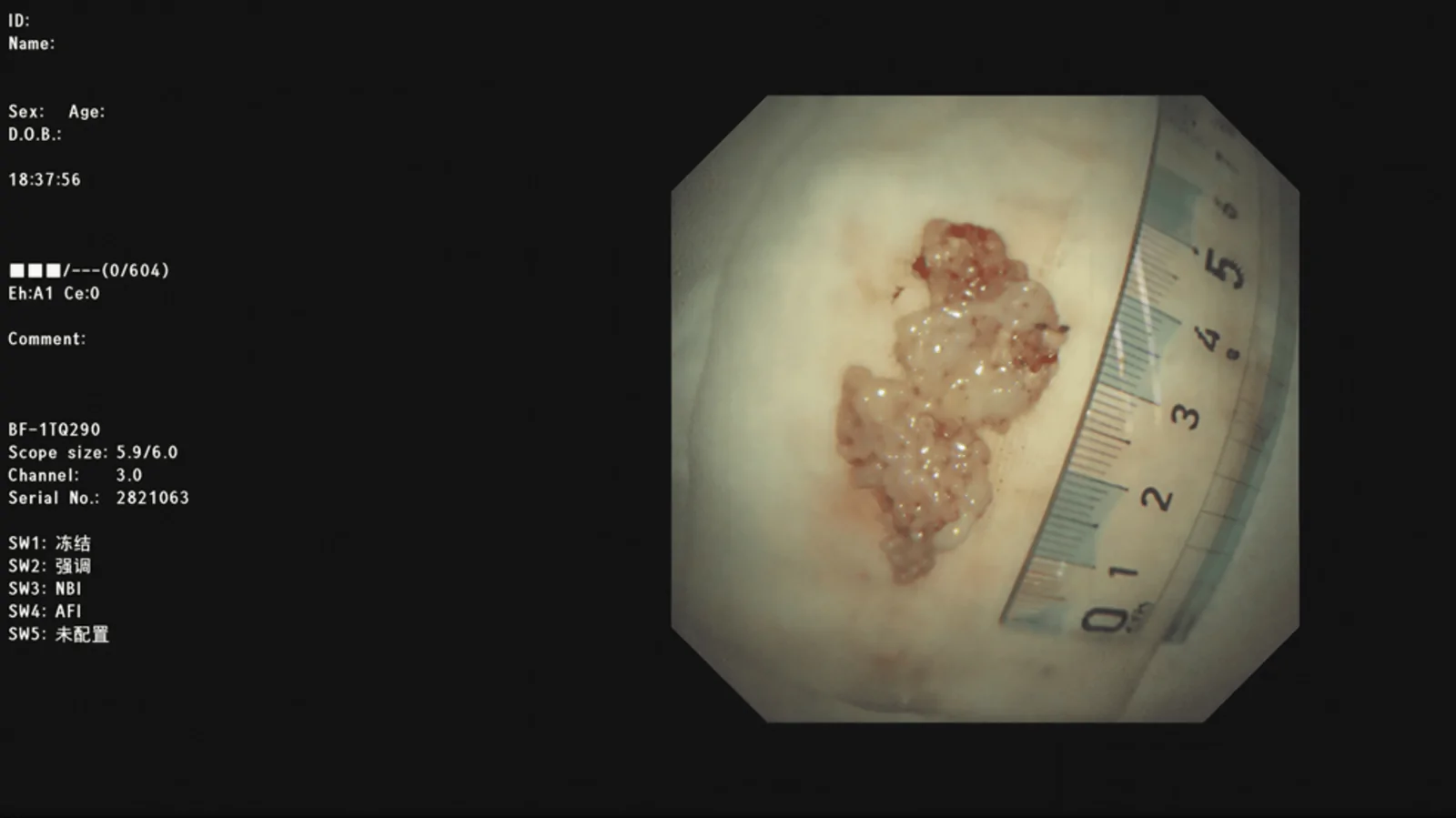
Resected tracheal tumor specimen measuring approximately 5 cm in length

Intraoperative vital signs remained stable throughout the operation, with heart rate ranging from 110 to 125 beats/min, blood pressure from 135 to 150/85 to 95 mmHg, and oxygen saturation maintained at 92-96%. No severe hypoxemia, sustained respiratory depression, or massive hemorrhage occurred. After tumor debulking, a 7.5‑mm endotracheal tube was placed under bronchoscopic guidance at a depth of 23 cm, and pressure‑controlled ventilation was initiated with a tidal volume of 7 mL·kg⁻¹, a positive end-expiratory pressure (PEEP) of 5 cmH₂O, a fraction of inspired oxygen (FiO₂) of 50%, and an inspiratory‑to‑expiratory ratio of 1:2, maintaining a partial pressure of end‑tidal carbon dioxide (PETCO₂) between 36 and 45 mmHg. At the end of the procedure, sedation and analgesia were discontinued. The patient was transferred to the intensive care unit with indwelling endotracheal intubation for mechanical ventilation, anti‑infective therapy, and cardiopulmonary supportive treatment.

On postoperative day 2, the patient met standard extubation criteria, including stable hemodynamics, no active airway bleeding, adequate spontaneous ventilation and oxygenation, clear consciousness with intact cough reflex, and negative cuff leak test excluding severe airway edema. The endotracheal tube was successfully removed, and high‑flow nasal oxygen therapy was administered. Follow‑up bronchoscopy on postoperative day 3 revealed no residual stenosis or recurrent bleeding. The patient's cardiopulmonary function and general condition gradually improved with continued symptomatic and supportive treatment.

## Discussion

Near-total tracheal stenosis caused by primary tracheal squamous cell carcinoma is a life-threatening difficult airway condition, in which minimal residual airway patency is prone to sudden occlusion and fatal asphyxia [4]. Emergency rigid bronchoscopic tumor debulking is the definitive rescue procedure, whereas perioperative management is extremely complex when superimposed on multiple high-risk conditions. The present patient harbored three synergistic risk factors: 96% severe tracheal stenosis, complete communication impairment due to congenital deaf-mutism and illiteracy without intraoperative family support, and hypoxia-induced myocardial injury. This triple high-risk profile substantially increased procedural difficulty, emphasizing the necessity of preoperative multidisciplinary consultation, a tiered rescue airway protocol, and individualized intraoperative strategies.

Complete verbal, written, and sign-language communication failure rendered the patient uncooperative, increasing the risk of intraoperative restlessness, unexpected body movement, and traumatic tumor hemorrhage during bronchoscopy [5]. To resolve this dilemma, we abandoned conventional awake-only management and adopted a cooperative-free sedative strategy combining graded topical anesthesia, low-dose intranasal dexmedetomidine (40 μg), and mild sevoflurane inhalation [6,7]. Intranasal dexmedetomidine provides stable, gradual sedation with negligible respiratory depression and does not require patient cooperation, which is uniquely suitable for emergency airway procedures in uncommunicative patients.

The patient's near-total fixed tracheal stenosis represented a critical central airway lesion with nearly exhausted ventilatory reserve, which contraindicated early deep sedation and neuromuscular blockade. Consistent with the 2022 American Society of Anesthesiologists difficult airway guidelines recommending spontaneous breathing preservation for patients intolerant of brief apnea, we implemented a rigorous two-stage sedation and ventilation strategy throughout the procedure [8]. During the pre-recanalization stage, only light sedation was maintained with spontaneous breathing strictly preserved to avoid loss of respiratory muscle tone and subsequent catastrophic airway collapse. Continuous bedside assessment of chest wall movement, breath sounds, and respiratory rate ensured stable spontaneous respiration during sevoflurane induction and HFJV, with no intraoperative apnea or hypoventilation observed. After tumor debulking reduced the stenosis degree from 96% to approximately 10%, secure airway patency allowed safe transition to positive-pressure ventilation. Stepwise deepened sedation with remimazolam and remifentanil was then initiated to suppress surgical stress and eliminate involuntary patient movement.

Given the patient's severe expiratory obstruction, critical stenosis, and concurrent type II respiratory failure, HFJV was selected as the optimal intraoperative ventilation modality. Proper parameter setting is critical to avoid HFJV-related complications, including hypoxemia, hypercapnia, barotrauma, and mucosal injury [9,10]. In this case, the low-to-moderate driving pressure of 0.25 MPa satisfied systemic oxygenation demand while preventing high-pressure airflow impact on fragile tumor tissues and injured airway mucosa, thereby reducing bleeding and barotrauma risks. A ventilation frequency of 60 cycles/min facilitated gas exchange via convection and diffusion, which adapted well to the severely stenotic airway geometry, whereas an inspiratory-to-expiratory ratio of 1:1.5 prolonged passive expiration to alleviate expiratory resistance, prevent gas trapping, and avoid carbon dioxide retention. To eliminate airway fire hazards during electrosurgical operation, oxygen supply was suspended for 60 seconds before argon plasma coagulation activation. Although intraoperative intraluminal oxygen concentration was not directly measured, this standardized low-oxygen protocol effectively minimized combustion risks during tumor ablation.

A hierarchical multidisciplinary rescue system was established preoperatively, with rigid bronchoscopic debulking as the first-line intervention, emergency tracheostomy as a limited backup for distal lesions, and VV-ECMO on standby for catastrophic airway failure. This graded protocol ensured comprehensive intraoperative safety. The patient's concurrent infection, renal impairment, and hypoxic cardiac injury reflected poor systemic reserve, further highlighting the value of our staged ventilation-sedation strategy in balancing surgical efficacy and respiratory/circulatory stability [11,12].

This study has several limitations. First, preoperative three-dimensional CT airway reconstruction was not performed due to emergency conditions, limiting precise anatomical evaluation. Second, continuous end-tidal carbon dioxide monitoring was unavailable during HFJV, hindering the early detection of carbon dioxide retention. Third, as a single retrospective case report, its generalization requires prudent caution. Further clinical accumulation is needed to optimize standardized anesthesia protocols for similar high-risk airway patients with communication disorders.

## Conclusions

This case demonstrates that for patients with near-total fixed tracheal stenosis combined with complete communication impairment, a two-stage anesthesia strategy with preserved spontaneous ventilation, graded topical anesthesia, and titrated HFJV is a feasible emergency approach. Preoperative formulation of a tiered multidisciplinary rescue airway plan is essential to cope with potential catastrophic airway events. Nevertheless, this single-case experience cannot be generalized. The lack of continuous intraoperative carbon dioxide and airway pressure monitoring remains an important limitation, and further clinical evidence is required to refine airway management protocols for similar high-risk patients.
